# To-Do and Not-To-Do in Model Studies of the Uptake, Fate and Metabolism of Metal-Containing Nanoparticles in Plants

**DOI:** 10.3390/nano10081480

**Published:** 2020-07-28

**Authors:** Justyna Wojcieszek, Javier Jiménez-Lamana, Lena Ruzik, Joanna Szpunar, Maciej Jarosz

**Affiliations:** 1 Chair of Analytical Chemistry, Faculty of Chemistry, Warsaw University of Technology, 3 Noakowskiego str., 00-664 Warsaw, Poland; jwojcieszek@ch.pw.edu.pl (J.W.); lenka@ch.pw.edu.pl (L.R.); mj@ch.pw.edu.pl (M.J.); 2 Universite de Pau et des Pays de l’Adour, E2S UPPA, CNRS, Institute of Analytical and Physical Chemistry for the Environment and Materials (IPREM), UMR 5254, 64053 Pau, France; joanna.szpunar@univ-pau.fr

**Keywords:** metal-containing nanoparticles, model plants, nano-bio interactions, transformations, physico-chemical characterization, mass spectrometry

## Abstract

Due to the increasing release of metal-containing nanoparticles into the environment, the investigation of their interactions with plants has become a hot topic for many research fields. However, the obtention of reliable data requires a careful design of experimental model studies. The behavior of nanoparticles has to be comprehensively investigated; their stability in growth media, bioaccumulation and characterization of their physicochemical forms taken-up by plants, identification of the species created following their dissolution/oxidation, and finally, their localization within plant tissues. On the basis of their strong expertise, the authors present guidelines for studies of interactions between metal-containing nanoparticles and plants.

## 1. Introduction

The extensive use of metal-containing nanoparticles (NPs) in an increasing number of applications is leading to their release into the environment, where they can interact with plants with unknown effects [1]. This interaction may lead to some impact on plant physiological processes and eventually to the bioaccumulation of NPs, and products of their metabolism, in the animal and human food chain. In the last years, the number of model studies focused on the investigation of interactions between plant and engineered nanoparticles, especially metal-containing ones, has increased [2,3,4]. For example, the bioaccumulation of silver nanoparticles (AgNPs) and aluminum oxide nanoparticles (Al_2_O_3_ NPs) in roots of *Lactuca sativa* L. followed by their translocation to shoots has been demonstrated [5,6]. A similar behavior has been observed during the study of the uptake and translocation of lead sulfide nanoparticles (PbS NPs), iron (III) oxide nanoparticles (Fe_2_O_3_ NPs) and magnetite nanoparticles (Fe_3_O_4_ NPs) in *Zea mays* L., *Triticum aestivum* L. and *Hordeum vulgare* L., respectively [7,8,9]. Contradictory results have also been observed for the same type of metal-containing nanoparticles in different plants: titanium oxide (TiO_2_ NPs) were not taken up by *Coriandrum sativum* L. [10], although their accumulation and translocation in tomato [11] or radish plants [12] has been reported. On the other hand, NPs may undergo different transformations after their accumulation in roots, followed by their translocation to above-ground organs as it has been observed for selenium nanoparticles (Se NPs) and AgNPs in garlic and Arabidopsis plants, respectively [13,14]. Finally, it is worth mentioning the accumulation of AgNPs in stems of three different tree species was faster after foliar treatment compared to roots treatment [15].

After uptake and accumulation, metal-containing NPs can interact with plants at the cellular and subcellular levels, facilitating changes to morphological and physiological states, which may be suppressive or stimulatory [2]. For instance, Ag NPs caused oxidative stress and exhibited toxicity when applied in higher concentrations to *Allium cepa* roots, regardless of surface coating used [16]. An increase of peroxidase, catalase, superoxide, dismutase activity, and inhibition in plant growth has been detected in *Lemna minor* after copper oxide nanoparticles (CuO NPs) treatment [17]. It has also been reported that the glutathione content and antioxidant power decreased significantly after *Trigonella foenum* cultivation with Al_2_O_3_ NPs [18]. On the other hand, metal-containing NPs may promote the plant growth and seed germination. For example, exposure of tomato to strong irradiance and TiO_2_ NPs resulted in better flower and fruit production, increased anthocyanin and carotenoids concentration and high enzyme activity although rapid chlorophyll content decrease was also observed [11]. The uptake and translocation of Fe_3_O_4_ NPs in *Hordeum vulgare* L. plants resulted in promoted gene expression and increase of some phenological parameters such as chlorophyll, total soluble protein and number of chloroplasts [9], although in *Eichhornia crassipes* plants a distinct decrease in chlorophyll content and catalase activity and an increase of malondialdehyde (MDA) content was observed after Fe_3_O_4_ NPs treatment at higher concentrations [19].

In this context, the comprehensive investigation of the behavior of metal-containing NPs throughout the whole process of interaction with plants—uptake, bioaccumulation, and translocation—is needed. However, such a challenge can only be accomplished through a careful design of experiments, where several factors must be taken into account, as well as through the use of several techniques that provide complementary information. The use of each technique will depend on the specific behavior of each metal-containing NPs (i.e., if they remain intact or they undergo dissolution and/or agglomeration) and thus on their chemical and physical nature. 

A number of analytical techniques is being currently used for the analysis and characterization of metal-containing NPs, such as transmission electron microscopy (TEM), scanning electron microscopy (SEM), atomic force microscopy (AFM), X-ray diffraction (XRD), energy dispersive X-ray spectroscopy (EDX), microbeam X-Ray Fluorescence (µ-XRF), microbeam X-ray absorption spectroscopy (µ-XAS) [3,20]. Microscopy based techniques are a commonly accepted characterization tool, TEM being the most widely used technique among them [4]. These techniques can provide information about particle size, shape, and agglomeration of metal-containing NPs accumulated by plants at the cellular and subcellular level [20]. However, the obtention of reliable data depends on statistical tools and a time consuming sample preparation based on a drying process, which in addition can lead to aggregation of NPs, especially in environmental samples [21]. On the other hand, synchrotron radiation (SR) based techniques have great potential to investigate localization and speciation of metal-containing NPs in plants. The combination of high-resolution synchrotron x-ray fluorescence microscopy (SR-XFM), offering multi-elemental detection down to the tens of nm, and spatially resolved XAS is a powerful technique that can provide information about elemental composition, localization and chemical speciation with minimal sample preparation and non-destructive analysis [22]. For instance, it has been successfully applied to the study TiO_2_ NPs [23,24], AgNPs [23,25], zinc oxide nanoparticles (ZnO NPs) [26,27] or cerium oxide nanoparticles (CeO_2_ NPs) [27] in different plants. The application of these techniques to the study of interactions between NPs and plants has been recently reviewed by Castillo-Michel et al. [22].

However, the majority of these techniques are inadequate for the characterization of NPs in complex matrices at low concentrations. In this context, the use of analytical methods based on the high-sensitive and element-specific technique of inductively coupled plasma mass spectrometry (ICP-MS) allows NPs detection at environmentally relevant concentrations. Indeed, an analytical tool that has become popular in these kinds of studies is Single Particle (SP) ICP-MS, thanks to a combination of the benefits of ICP-MS, with those of a particle counting technique [28]. SP-ICP-MS can provide information about the particle size, particle size distribution as well as quantitative information about the metal in its dissolved and nanoparticulate form in a single analysis. Typically, the analysis of a NP solution by SP-ICP-MS produces a time scan with two different types of signal: a steady signal at low counts due to the background or the presence of the analyte in its dissolve form; and a number of pulses above the background due to the presence of the analyte in its nanoparticulated form [29]. 

**Figure 1:**
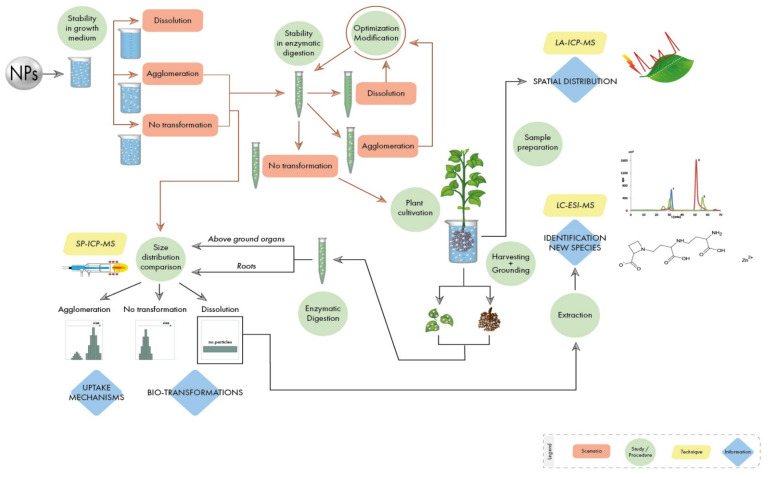
Flowchart presenting possible scenarios and steps to be carried out during studies of nanoparticles (NPs)–plant interactions.

Authors present here a step-by-step guideline in order to obtain reliable data in metal-containing NPs-plant interaction studies, including: the study of the stability of NPs in nutrient solutions used for plant cultivation; the optimization of a procedure able to extract NPs from the plant matrix without altering their properties; the analysis of NPs in plant tissues by monitoring their possible transformations; the identification of new metal species created within the plant as a result of dissolution/oxidation processes; the study of the spatial distribution of NPs in plant tissues. A flowchart with the necessary steps in these kinds of studies is shown in Figure 1. Authors use their extensive experience with the investigation of NPs of different chemical nature and properties to explain the different scenarios stressing some critical points where special attention needs to be paid. It must be stated that the guidelines described here apply to the experimental scheme shown in Figure 1, through the use of different analytical techniques based on mass spectrometry, which has proven to give comprehensive information. However, it must be highlighted that, besides the general rules draw in this manuscript, each type of metal-containing NP has specific properties (size, shape, type of metal, coating, etc.) and will require an individual study.

## 2. Transformation of Metal-Containing Nanoparticles in Growth Media 

Model studies carried out in order to get deeper insight about metal-containing NPs interactions with plants include several steps. The first one involves the growth of plants in the presence of metal/metal oxide NPs in a soil environment or in a hydroponic solution, being the latter the most used in literature. Different media can be used for plant cultivation, for example, Murashige and Scoog, Hoagland or Knop nutrient solution [30,31].

The composition of the nutrient solution may have a negative impact on the interpretation of the results and may need to be adapted or modified. On the one hand, and due to the high sensitivity of the analytical techniques used in this kind of study, the presence of the metal of interest even at low concentration in the medium may lead to the occurrence of false positives when it comes to the analysis of plant tissues. For instance, salts usually present in growth media, like ZnSO_4_, CuSO_4_ or FeSO_4_, should be removed during the analysis of ZnO, CuO or Fe_2_O_3_ NPs, respectively. A possible alternative could be the use of isotopically labeled metal NPs, which would allow the discrimination from different metal sources. Isotopic labeling of NPs enables their sensitive tracing in the presence of background elements in complex plant matrices [32]. On the other hand, special attention has to be paid if metal compounds with complexing agents such as ethylenediaminetetraacetic acid (EDTA), iminodiacetic acid (IMDA) or diethylenetriaminepentaacetic acid (DTPA) are added to a growth solution. For example, the presence of EDTA, a very strong ligand, can bind metal ions potentially released from the analyzed NPs and shift the equilibrium in the system, leading hence to biased results. In addition, the identification of metal-EDTA complexes created as a result of metal releasing from NPs will not be useful information as this kind of compound cannot be formed inside plant tissues. In this context, Fe-EDTA present in growth media like Knop nutrient solution must be replaced by another salt, like FeSO_4_ [33]. 

Metal-containing NPs are reactive species that can interact with the surrounding environment. In this context, the stability of NPs in the growth medium used for plant cultivation needs to be investigated before the cultivation and analysis of plants. These studies are usually performed by spiking the nutrient solution with a suspension of metal-containing NPs followed by its analysis immediately upon addition of the NPs suspension and over time. The last analysis should be performed at the endpoint time of the subsequent plant cultivation. This previous study is critical in model studies of NPs-plants interaction in order to elucidate if the possible transformations of NPs take place before or after their uptake by plants. In general, there are three possible scenarios according to the transformations that NPs can undergo: (i) NPs remain unchanged in medium; (ii) NPs are partially or totally dissolved; and (iii) NPs agglomerate over time. The occurrence (or not) and extent of these transformations will depend on the nature of the NPs used and requires an individual and specific study. For this purpose, SP-ICP-MS is a technique that can provide information about NP size distribution and the physico-chemical form of the metal of interest in a single analysis, which makes it an ideal tool to monitor possible NP transformation in growth medium. Examples of application of SP-ICP-MS to the characterization of metal-containing nanoparticles in hydroponic solution under the three mentioned scenarios are described below. 

The simplest situation that may happen during the study of the stability of NPs in a nutrient solution is that they are stable during the whole cultivation period. In that case, the obtained size distribution must be in good agreement with that obtained for the stock suspension [34]. Additionally, in SP-ICP-MS, the absence of a high background signal (corresponding to the metal in its dissolved from) and pulses of high intensity signal (corresponding to NPs of bigger sizes) will prove that NPs do not undergo any transformation such as dissolution or agglomeration in nutrient solution used for plant cultivation. As a consequence, any NPs transformation detected during the analysis of tissues of treated plants, must be attributed to processes taking place inside plant tissues, after the uptake and accumulation of NPs. 

However, growth medium can cause the dissolution of metal-containing NPs. It can happen especially in the case of microelements that are present in high abundance in natural environments, such as zinc or copper, usually present in different forms such as free ions, compounds with different bioligands or as a component of rocks. This phenomenon can be easily identified thanks to the use of SP-ICP-MS. In this case, on the time scans obtained during SP-ICP-MS analysis of growth medium, a steady signal, i.e., characteristic of the dissolved form of the metal, is registered, whereas a significant number of pulses proving the presence of NPs is observed after analysis of fresh NPs stock suspension. If only a steady signal is observed after analysis of growth medium, it leads to the conclusion that plants are mainly taking up metal in its dissolved form and therefore the accumulation of NPs is negligible. In the case of the presence of only the dissolved form of metal, speciation studies leading to the quantification and identification of metal complexes formed inside the plant tissues must be carried out, as it is explained in detail in Section 6 of this manuscript. The dissolution of NPs is time dependent and can be partial, with both dissolved and particulate form of metal present in the growth medium. In this case, if both forms of metal are taken up by plants, both NP characterization by SP-ICP-MS as well as speciation studies by the use of hyphenated techniques should be performed. If only metal ions are taken up by plants, then characterization of NPs is not possible and the direction of the study has to be changed. The dissolution rate of NPs strongly depends on media composition as well as on the surface coating of NPs. It has been shown in different studies that bare nanoparticles are more susceptible to transformation than coated NPs [35,36]. 

In the last scenario, NPs can undergo agglomeration as it was observed for some nanoparticles based on metal oxides [12,37]. In this case, the extent of the agglomeration must be determined since it may have an influence on the potential uptake by the plant. For instance, it can happen that the median diameter of NPs shifts slightly toward bigger sizes after different times of incubation or that NP size duplicate or triplicate, which leads to the conclusion that NPs can undergo agglomeration in a smaller or bigger degree after different contact time with growth medium. In any case, the critical point is to determine whether agglomerates of NPs created in a nutrient solution are taken up by plant tissues. Typically, only NPs at smaller sizes than those observed in the growth medium are accumulated in plant leaves and roots [12,37,38]. The occurrence of agglomeration processes can be avoided by the use of coated-NPs and/or by the addition of some additional reagents such as organic acids or enzymes to the solution used for plant cultivation. The former option is clearly advised, since the latter may be problematic from the point of view of mimicking natural conditions and should be avoided as much as possible. 

As it has been described on the three scenarios, is it important to monitor the possible transformation processes of NPs in growth medium used during plant cultivation. The use of SP-ICP-MS can provide clear information on whether NPs are stable or if they undergo different transformations such as dissolution or agglomeration in a single analysis.

Finally, it should be mentioned that the studies of the interaction between metal-containing NPs and plants can also be performed in solid media, in order to mimic natural conditions, where NPs are released into soil environments. Therefore, the stability of metal-containing NPs in a soil environment must be investigated, not only to investigate the uptake by plants but also to understand the terrestrial toxicity of NPs [39]. However, these studies are less convenient than those performed in a liquid medium, since they imply an additional sample preparation step: the extraction of NPs from the solid medium without changing their physico-chemical form. In this context, extractions with purified water and with tetrasodium pyrophosphate (TSPP), with sonication to enhance particle dispersion, followed by analysis by SP-ICP-MS have been proposed [40,41]. The type of media (liquid or solid) used for plant cultivation may have a significant influence on NPs properties. For example, the bioavailability and the effect of the silver ions released by AgNPs have been shown to be lower in a soil medium compared with an agar medium [42]. In any case, regardless of the medium used for plant cultivation, the fate and possible transformations of NPs must be monitored.

## 3. Sample Preparation and Total Content Determination 

After an investigation of NPs stability in nutrient solution, the cultivation of plants in the presence of NPs and control plants is the next step to be carried out. Afterward, plants are divided into different organs/tissues and subsequently grounded. The use of a pestle and mortar is highly recommended. The translocation factor from roots to above-ground organs can be easily calculated at this stage, by determining the total content of metal within the different plant organs. For this purpose, mineralization processes with oxidizing acids and heating systems or microwave-assisted techniques are commonly used for acid digestion of organic matrices [43], followed by quantitative analysis by standalone ICP-MS or inductively coupled plasma optical emission spectrometry (ICP-OES). The choice of the acid(s) used for the digestion of plant tissues will depend on the nature of the metal the NPs are made of. Some metal-containing nanoparticles can dissolve under acidic conditions, mainly by the use of concentrated nitric acid, but others will require different or additional reagents: aqua regia for platinum nanoparticles (PtNPs) and palladium nanoparticles (PdNPs) [34,38]; hydrogen peroxide for ZnO NPs and CeO_2_ NPs [33,37]; or hydrofluoric acid for TiO_2_ NPs. 

## 4. Extraction of Intact Nanoparticles 

Although analysis of total metal content provides a general idea of the metal bioaccumulation by plants, when it comes to studies with metal-containing NPs, important information like the physico-chemical form, the size distribution or the nanoparticle number concentration is lost after acid digestion. The correct interpretation of the data strongly depends on the extraction of NPs from the plant matrix preserving their native conditions. Therefore, the first important step in the analytical procedure is to develop and optimize an efficient extraction process of NPs from plant material. 

Plant tissue matrix is generally made of some or all of the following components: macro and micronutrients, vitamins, amino acids or other nitrogen supplements. In addition, the plant cell wall is composed primarily of polysaccharides, cellulose being its major component, and it is organized into paracrystalline structures inserted in a rich matrix of diverse polysaccharides, including hemicelluloses and pectins, structural glycoproteins and lignin in certain tissues [44].

NPs can penetrate through the cell wall, so the digestion of polysaccharides is needed (for example, pectin consists of four major polysaccharide domains: homogalacturonan (HGA), rhamnogalacturonan I (RGI), rhamnogalacturonan II (RGII) and xylogalacturonan (XGA)) with the preservation of the NPs at the same time. This can be done through the use of alkaline solutions or enzymes, for example, pectinase, hemicellulase, and cellulase, which are perfectly suited for breaking down the polysaccharides found in plant cell walls. Alkaline treatments with tetramethylammonium hydroxide (TMAH) have shown a high-efficiency extraction of different NPs (Ag, Au, or carbon nanotubes) from tissues with a low amount of salts remaining in solution after digestion [45]. It should be mentioned that an alkaline treatment followed by SP-ICP-MS analysis could not be used for extracting AgNPs from tissue samples. This is important information regarding the changes in the state of AgNPs, most probably due to Ag^+^ precipitation and/or AgNPs aggregation [46]. 

Enzymatic digestion, which commonly works with mild conditions, i.e., at moderate temperatures and pH conditions, can be an appealing sample pre-treatment for isolating NPs without their degradation. The use of a multi-component enzyme mixture containing cellulase, hemicellulase, and pectinase (Macerozyme R-10) was proposed by Dan et al. for isolating gold nanoparticles (AuNPs) from roots of tomato plants [47]. In addition, a suitable extraction method should not only extract intact NPs but also a representative amount of them. For this purpose, the mentioned enzymatic digestion method was further developed by Jimenez-Lamana et al. [34]. As it was demonstrated in this study, different parameters (type of buffer used, amount of sample, amount of enzyme, sonication power, sonication time, incubation time) need to be studied and optimized to obtain the highest number of NPs from the different plant tissues.

**Figure 2:**
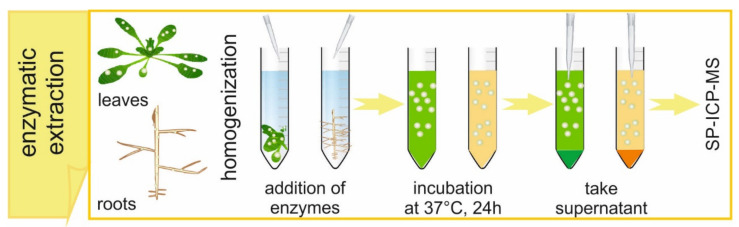
Steps to be performed in a typical enzymatic digestion procedure.

A typical enzymatic digestion procedure for the extraction of metal-containing NPs from plant tissues is shown in Figure 2. It is important to highlight the use of well-grounded samples in order to provide the maximum physical contact between sample and reagents. Ground samples are mixed with citrate buffer and the mixture is next homogenized using an ultrasonic probe while the tube is kept in ice. Milder procedures, like bath sonication or shaking, should be avoided, since they do not provide a successful extraction of NPs from plant tissues. On the other hand, nominal powers of a probe higher than 35% are not recommended, to avoid excessive heating of the sample. After the end of homogenization, the enzyme solution is added. The samples are shaken in a water bath at 37 °C for 24 h. After the incubation, the obtained suspensions are filtered with a 0.45 µm syringe filter because of the presence of a remaining solid after homogenization and incubation.

Important precautions must be taken depending on the nature of the metal-containing NPs object of the study. For instance, the sonication probe may leach significant amounts of titanium into the suspension which, even at trace levels, may lead to the contamination of the plant samples and hence the occurrence of false positives in studies of TiO_2_ NPs interactions with plants [12]. To avoid that, a tissue grinder set can be used instead.

The effect that the digestion procedure may have on NPs (dissolution and/or aggregation) must be investigated. To do so, the same procedure must be performed on a suspension of the metal-containing NPs followed by its analysis. As indicated above, the characterization and monitoring of metal-containing NPs and the processes they may undergo can be easily carried out by SP-ICP-MS. In one single analysis, SP-ICP-MS can provide the necessary information to decide if the chosen digestion protocol is suitable for extracting NPs from the plant tissues without altering their properties. Finally, the influence of the plant matrix can be additionally investigated by submitting control plant tissues (i.e., cultivated in the absence of NPs) spiked with a suspension of metal-containing NPs to the digestion procedure, obtaining the corresponding size distributions by SP-ICP-MS and comparing with the original NP size distribution. 

It is important to mention that the use of a filtration step after the digestion procedure could imply the loss of bigger NPs [12], leading to unreliable results. In that case, filtration should be discarded and it is advisable to let the suspensions settle down after digestion for at least one hour and take the supernatants to analyze. The use of centrifugation at this step is not recommended since NPs will also settle down to the bottom of the suspension.

## 5. Uptake, Translocation and Biotransformation 

Once the stability of the metal-containing NPs suspension in the growth media and the suitability of the digestion procedure has been investigated, the plant cultivation in the presence of NPs must be carried out. During this process, a complementary evaluation by plant scientists should be carried out, i.e., the tolerance of the plant to the NP concentration in terms of phytotoxic effects. Different factors, such as color of plant tissues, biomass production or tissue hydration can be determined and compared with those obtained for control plant samples. If possible, detailed tests for eventual phytotoxicity on the cellular level should be undertaken. 

As it was mentioned above, the total content of metal taken up by the plant does not provide information about the form of element accumulated in plant tissues or about possible transformations of NPs that can happen during uptake and transport. In this context, and in order to investigate the physico-chemical form of metal accumulated inside plant tissues, SP-ICP-MS can be a valuable tool. It is important to mention that in SP-ICP-MS, the dilution of the sample plays an important role, in order to be able to detect signals produced by individual NPs. Typically, samples with NP concentration around 1 × 10^8^ NP L^-1^ are analyzed in SP-ICP-MS. This is especially important in the case of roots, where higher NPs concentrations are expected. Different scenarios can be considered during SP-ICP-MS analysis, depending on several factors such as the chemical nature of the metal-containing NPs, type of plant or conditions of cultivation. 

Similarly to the investigation of metal-containing NPs stability in the growth media, unchanged NPs can be taken up and accumulated in plants, i.e., no transformations such as agglomeration or dissolution take place. This can be easily observed by a simple comparison of the size distributions obtained in plant tissues with the one obtained for a fresh suspension of the metal-containing NPs. The presence of NPs in both roots and leaves presenting the same size distribution and nominal diameter as the stock NPs suspension means that analyzed plant has an ability not only to uptake and accumulate NPs in roots but also to translocate them to above ground organs. 

A different situation can be found when metal-containing NPs undergo agglomeration inside plant tissues [34]. This phenomenon can be clearly seen when observing the size distribution obtained by SP-ICP-MS. If the agglomeration process occurs, two populations are observed on the size distribution: a main one at sizes close to the nominal diameter, corresponding hence to unchanged NPs; and a second population at larger sizes not observed during the analysis of stock suspension. In order to elucidate at which step of the plant cultivation the metal-containing NPs undergo agglomeration, the particle size distribution obtained in the roots must be compared with the one obtained previously in the stability study in growth media. If no agglomeration is observed in the nutrient solution spiked with NPs, the presence of second distribution at higher sizes will imply that NPs undergo agglomeration during their uptake.

On the other hand, agglomeration can already occur in a nutrient solution used for plant cultivation. Again, a comparison between NP size distributions obtained in plant tissues and those obtained in the growth medium spiked by NPs over time must be performed. The key question here is whether agglomerates eventually created in a nutrient solution are taken up by plant tissues. For example, only smaller NPs may be taken up by plant roots, followed by the transport of intact NPs to stems and leaves [12], suggesting that agglomerates are not taken up by plant tissues. This can be easily recognized by a simple comparison of size distributions, since if only smaller NPs are taken up by plants, size distributions obtained in roots after plant cultivation will present particles with lower diameters in comparison with analysis of NPs suspension in growth medium. However, it can also happen, despite the fact that only smaller NPs are taken up by roots, that bigger NPs are found in the analysis of leaves and stems [37]. In this case, the results suggest that NPs can also undergo agglomeration at the endpoint of their transportation, where nanoparticles are more locally concentrated and hence agglomeration by contact is more likely to occur. The uptake of metal ions followed by re-precipitation can also be considered as a pathway of NPs accumulation in plants, which was already suggested during analysis of CeO_2_ NPs in three plant species by scanning transmission electron microscopy (STEM) [48]. 

Finally, the dissolution of NPs can also be observed after SP-ICP-MS analysis, which resulted in a steady signal on time scans, without pulses characteristic for particulate form of metal. Similarly to the case of agglomeration, dissolution can appear at different stages of the experiment. For instance, partial dissolution of metal-containing NPs may occur inside plant tissues after their uptake as intact NPs [38], which brings the opportunity to study both NPs characterization by SP-ICP-MS together with metal speciation in plant tissues by the use of separation techniques coupled to mass spectrometry detection [38].

**Figure 3:**
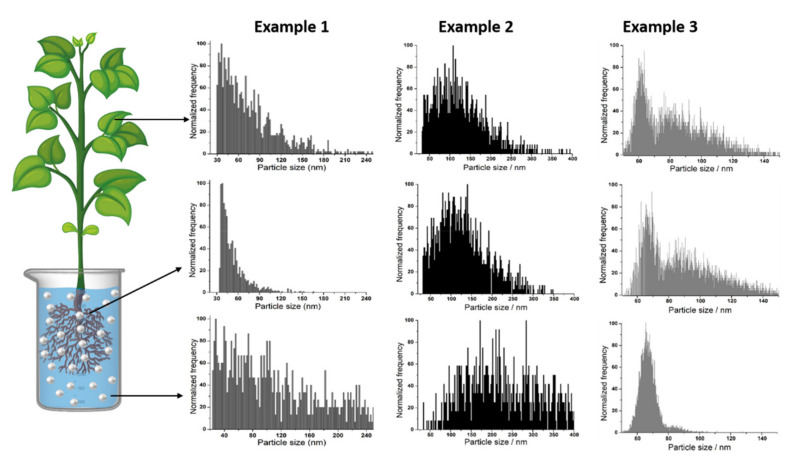
Comparison of size distributions obtained for metal-containing NPs in growth medium, roots and leaves in 3 different scenarios.

Figure 3 reflects how a simple comparison of size distributions obtained by SP-ICP-MS of metal-containing NPs in growth medium, roots and leaves can provide useful information. Example 1 shows the case of NPs that agglomerate in growth medium, only smaller ones are taken up by the plant and a re-agglomeration process occurs during transport to leaves. In example 2, agglomerates are not taken up from medium either but NPs remain intact during transport. Finally, in example 3, NPs remain unchanged in growth medium but they undergo agglomeration during uptake and/or transport. 

## 6. Speciation Studies

As it has been mentioned, metal-containing NPs may undergo partial or total dissolution in the nutrient solution, leading to the uptake of metal ions by roots [33]. The analysis of the total metal content in plant organs can provide preliminary conclusions about the physico-chemical form of NPs, as the translocation factor of analyzed metal from roots to leaves is usually significantly higher if dissolved metal is transported within the plant. If the dissolution of the metal-containing NPs in growth medium is suspected, the cultivation of an additional set of plants treated with corresponding metal salt at the same metal concentration is recommended. In this case, the comparison of total metal content in plant tissues treated with metal salt and metal-containing NPs can give additional information about the uptake mechanisms. In any case, and regardless of whether a dissolution process takes place already in the growth medium used for plant cultivation or inside plant tissues after NPs uptake and accumulation, the presence of the dissolved form of metal redirects the investigation towards another direction. 

The key point is that the presence of the metal in its free form may lead to the formation of new species inside the plant and hence studies on metal speciation to determine and identify these new species are needed. For this purpose, the extraction of metal compounds formed in plant tissues must be carried out in order to release them from solid matrix. If one-step extraction turns out to be insufficient, additional steps should be applied, for example, by the use of enzymes such as pectinase or cellulase. The use of these enzymes leads to an increase of extraction efficiency by degradation of the plant cell wall, which allows the release of metal accumulated in this part of the tissue. The efficiency of extraction can be determined by the digestion of each extract followed by metal content analysis and comparison with the total metal concentration. It should be highlighted that, due to its different chemical nature, procedure of extraction of metal compounds must be different than the digestion procedure used for extraction of intact NPs from plant tissues. 

Speciation analysis can be successfully performed by the use of hyphenated techniques, based on the coupling of effective separation technique such as chromatography or electrophoresis to ICP-MS for sensitive element-specific detection or/and to mass spectrometry with soft ionization (electrospray (ESI) MS, matrix-assisted laser desorption/ionization (MALDI) MS) for identification of a compound structure. For analysis of complicated matrix such as plant samples, an initial fractionation of extracted compounds in terms of molecular mass can be performed by size exclusion chromatography (SEC) coupled to ICP-MS. SEC is used for simple fractionation of metallocompounds according to their molecular mass. In addition, SEC analyses are performed under mild conditions and without the use of organic solvents, which is important to maintain the stability of metal species. However, each peak obtained during SEC-ICP-MS analysis contains a significant number of metal compounds, since resolution of SEC is rather low. Therefore, prior to the identification of extracted complexes, a different technique that allows better separation of initially fractionated species, should be applied. One of the possible options is that after SEC-ICP-MS analysis, fractions corresponding to each peak observed on the chromatograms can be collected, lyophilized and analyzed by hydrophilic interaction chromatography (HILIC) coupled to ICP-MS [7], which provides a more efficient separation of metal compounds, in terms of their polarity. Results obtained by using ICP-MS as an elemental detector provide information about type and nature of extracted metal complexes, without information about the ligands bound to the analyzed metal. Therefore, after characterization of the extracted metal species by means of two-dimensional liquid chromatography coupled to ICP-MS, identification of the compounds formed in plant tissues must be performed. For this purpose, tandem mass spectrometry (MS/MS) with soft ionization such as ESI or MALDI is usually used by fragmentation of molecular ions and detection of specific fragment ions. It can be performed by direct analysis of samples or after their separation using chromatographic techniques. However, in the case of matrices that contain many different compounds, the results obtained by direct MS/MS analysis can be complex to interpret. In order to identify extracted metal complexes, HILIC chromatography coupled to high resolution electrospray tandem mass spectrometry (ESI-MS/MS) is highly recommended to use. HILIC chromatography is well suited to mass spectrometry detection, as typical eluents used in this separation technique contain a high percentage of organic solvent in water or volatile buffer. The high resolution of state-of-the-art ESI-MS together with high mass accuracy allow for automatic data mining in search for metal species and organic compounds, based on their isotopic patterns and species mass defects. The mass spectrum registered during analysis of extracted metal compounds must be searched for parent ions with a specific isotopic pattern corresponding to analyzed metal (unless it is monoisotopic) and chosen ions should be fragmented next for determination of species structure. Collision energy should be optimized during analysis since it may occur that fragmentation is too strong or not strong enough and contribution of the parent ion is too big. Thanks to this approach, the identification of extracted complexes can be confirmed in terms of molecular mass, MS/MS fragmentation and by matching of the registered isotopic patterns with the theoretical ones. If there is one major metal compound observed, the quantification can be carried out by an external calibration curve and/or standard addition method.

Finally, it should be highlighted that the use of hyphenated techniques for metal speciation in various matrices allows the detection and identification of even minor metal complexes at environmentally relevant concentrations.

## 7. Spatial Distribution

The detection and characterization of NPs in above ground tissues demonstrates the ability of plants to take up and translocate NPs. However, the exact pathways and mechanisms of uptake and transport of NPs are still unclear. For example, damage of the physiological barriers of roots [4] or penetration into the root epidermis and cortex through the apoplastic pathway have been suggested [49]. In this context, the study of the spatial distribution of metal-containing NPs in plant roots can help in filling this gap. 

The analytical techniques described so far are not able to provide this information. However, Laser Ablation (LA) ICP-MS has proved to be a valuable analytical tool for bioimaging of metal species in plant tissues [50]. This technique is based on the vaporization of a solid sample by using pulses from a focused laser beam, the ablated material being transported to the ICP-MS in a gas flow of Ar or He [51]. Even though LA-ICP-MS is not able to provide information about the physico-chemical form of the metal analyzed, it can be successfully applied when the information about the presence of the metal in its nanoparticulate form has been confirmed by another technique [37]. Sample preparation is a critical step for the analysis of plant organs by LA-ICP-MS. Usually, thin layers of the plant tissue are prepared with a microtome, which are directly mounted into glass slides. However, the sample preparation procedure will depend on the shape and geometry of the analyzed organ. The analysis performed by LA-ICP-MS can provide useful information about the localization of NPs inside the tissue that will help to elucidate the uptake and transport mechanisms. For instance, the accumulation and transport of CeO2 NPs by secondary roots from the bottom towards the central part of radish roots was suggested after analysis by LA-ICP-MS [37]. 

LA-ICP-MS can also provide quantitative information but requires the use and availability of matrix matching standards to constitute calibration curves and appropriate internal standards (IS) to compensate signal variation during laser beam-sample interaction, transportation of the aerosol, and instrumental drifts [52,53], making the analyses more complex. In addition, the use of a single imaging technique like LA-ICP-MS may result insufficient in studies at low concentrations, where the few observable NPs can be almost indistinguishable from naturally occurring NPs or other background signals [54]. 

It is then advisable, when possible, to combine the information obtained about spatial distribution of metal nanoparticles by LA-ICP-MS, with the information obtained with other analytical techniques: total metal determination (ICP-MS) and characterization of physico-chemical form (SP-ICP-MS).

## 8. Conclusions

The investigation of interactions between metal-containing NPs and plants requires several steps that need to be carefully designed, each of them requiring a careful and individual evaluation depending on the plant used in a model study, the cultivation conditions and especially the type of metal NPs studied. In addition, for a correct characterization of metal-containing NPs at each step and/or identification of new species created, a multi-technique approach is needed. Here we summarize the most important recommendations developed along the text:

- The behavior of metal-containing NPs in the nutrient solution used for plant cultivation must be investigated before starting the cultivation experiment. The occurrence of a transformation process in the growth medium (agglomeration/dissolution) may influence the rest of the study.
- NPs must be extracted from the plant material without altering their properties. The use of an enzymatic digestion with an enzyme capable of digesting plant cell walls, like pectinase, cellulase or hemicellulase is necessary. A cocktail of enzymes (e.g., Macerozyme R-10) is recommended.
- SP-ICP-MS has proved to be a useful tool for size characterization of metal-containing NPs in plant tissues as well as to monitor possible NPs transformation inside the plants.
- The comparison of NPs size distributions in the nutrient solution, roots and above-ground organs provides essential information about the mechanisms of uptake and translocation of metal-containing NPs by plants.
- If NPs dissolution takes place, speciation studies leading to the identification of new metal species created within plant tissues can be carried out by hyphenated techniques, which include separation of extracted compounds by chromatography or electrophoresis, followed by their characterization and/or identification by mass spectrometry.
- Additional information about the spatial location of metal following the uptake of NPs in tissues can be obtained by laser ablation coupled to ICP-MS. This, in combination with the information obtained about the physico-chemical form of NPs inside plants, can provide new insights about the uptake and translocation pathways.
